# Prevalence and Correlates of Intimate Partner Violence against Women in Liberia: Findings from 2019–2020 Demographic and Health Survey

**DOI:** 10.3390/ijerph19063519

**Published:** 2022-03-16

**Authors:** Masood Ali Shaikh

**Affiliations:** Independent Researcher, Karachi 75300, Pakistan; masoodalishaikh@gmail.com

**Keywords:** intimate partner violence, women, Liberia

## Abstract

Background: Intimate partner violence is a global public health crisis and a human rights issue. The objectives of the study were to conduct secondary analysis of the most recent Liberia Demographic and Health Survey (2019–2020) to determine the descriptive and analytical epidemiology of intimate partner violence (IPV) and its correlates in 15–49 year old ever-married women. Methods: Association of physical, emotional, sexual, and having experienced any type of IPV with 10 explanatory socio-demographic, attitudinal, and experiences were analyzed using simple and multiple logistic regression models. Results: 55.29% of women reported having ever experienced some form of IPV perpetrated by their current or most recent husband/partner, with the most common type being physical violence. Having been slapped, insulted, made to feel bad, and physically forced into unwanted sex were the most common types of physical, emotional, and sexual IPV. The multivariable analysis showed statistically significant association with IPV for number of living children, women’s acceptance of IPV, husband/partner’s use of alcohol, and having witnessed parental physical IPV. Conclusions: The prevalence of having experienced physical and/or sexual intimate partner violence in Liberia was much higher than the prevalence for the WHO Africa region of 33%, highlighting the need for better women empowerment and gender equality in Liberia.

## 1. Introduction

Intimate partner violence (IPV) has been a socially acceptable practice since antiquity [1,2,3]. As early as 753 BC, the Roman ruler Romulus promulgated ‘The Laws of Chastisement’, sanctioning wife beatings with a stick no thicker in circumstance than the man’s right thumb, i.e., the rule of thumb [1]. Physical and economic power inequality between the two sexes and historically sanctioned view of owning one’s women have been the IPV conduits [2,3]. IPV is a serious global public health problem and a human rights violation. IPV is defined by the World Health Organization (WHO) as “any behavior within an intimate relationship that causes physical, psychological, or sexual harm to those in the relationship” [4]. 

Lifetime physical and sexual IPV prevalence in ever partnered women aged 15–49 years were reported to be 27% globally, and 33% in WHO Africa region [5]. Myriad demographic, social, and cultural risk factors were identified in single country, multiple country, and meta-analyses studies for higher IPV prevalence against women, that include younger age, low household income, low educational attainment of women, having witnessed parental violence and intergenerational violence transmission, partner’s alcohol use, acceptance of violence by women, and rural residency status [6,7,8,9,10,11,12,13,14,15]. Notably, two important meta-analyses were recently reported that looked at selected risk factors associated with IPV. A meta-analysis from 25 sub-Saharan African countries using Demographic and Health Survey (DHS) data reinforced the association of increased IPV prevalence with rural residency status, poor living conditions, and low educational attainment in women [7]. Another meta-analysis from 44 countries that included 29 countries from sub-Saharan Africa using DHS data also reported similar higher IPV prevalence with low educational attainment in women and rural residency status [15]. 

The Republic of Liberia forms the west African coast, with a population of about five million, and had the Human Development Index rank of 175 out of a total of 189 countries in 2019 [16]. A coup in early 2003 lead to the ‘United Nations Mission in Liberia’ for providing security. This was followed by an election of a president in 2011; and in the 2017 election, a new president was elected that marked an internationally recognized democratic transition in Liberia after almost three quarters of a century. However, civil strife and resultant political upheavals in the first decade of the new century took its toll in terms of increased IPV. No nationally representative studies are available on the prevalence and correlates of IPV in Liberia since 2007. However, few studies exist on the association between IPV and political and armed conflicts. Exposure to violence in one’s communities and food insecurity during and in the aftermath of these conflicts were reported to increase IPV prevalence [17,18]. 

The objectives of this study were to conduct secondary analysis of the most recent Liberia Demographic and Health Survey to determine the descriptive and analytical epidemiology of IPV and its correlates in 15–49 year old ever-married women. 

## 2. Materials and Methods

### 2.1. Study Area and Data Source

This secondary analysis was based on 2019–2020 cross-sectional, Liberian demographic and health survey (LDHS) data. The data collection phase was started in October 2019 and completed in February 2020. Liberia is administratively subdivided into 15 counties and 136 districts, with each district further subdivided into clans. For the last census in 2008, each clan was further subdivided geographically into enumeration areas (EA) that comprised of an average 100 households. LDHS used stratified two stage cluster sampling design. In the first stage, using probability proportional to size method, 325 clusters comprising of EAs were selected. In the second stage, households were listed in each cluster, and using equal probability systematic sampling method, 30 households were selected from each cluster. This resulted in the selection of 9745 households for 2019–2020 LDHS, based on 2008 census and subsequent population projections. All 15–49 year old women in these selected households were deemed eligible for the LDHS. However, in adherence to the World Health Organization’s guidelines on ethical conduct of collecting domestic violence data, only one randomly selected woman in a subsample of households was administered a domestic violence module, while ensuring privacy [19]. This is the common survey methodology used in the DHSs for the implementation of a domestic violence module. The ‘Domestic Violence’ module in Liberia was administered to a subset of all women who were interviewed. In each household, one woman was randomly selected for this module. 

The approval for secondary analysis of LDHS was granted by Measure DHS, using an online request form; datafile was downloaded from the Measure website www.measuredhs.com (accessed on 20 August 2021). LDHS covered all 15 states and cumulatively had 8364 eligible women aged 15–49 years. Out of these, 8065 (96.43%) women were interviewed. For the domestic violence module, 3166 women aged 15–49 years were selected, but 46 (1.45%) women could not be interviewed owing to lack of privacy, inability to find respondents at home even after repeated visits, or due to interruptions during the interview. Hence, domestic violence was administered to 3120 women who were successfully interviewed. Out of these 3120 women, there were 2331 women who were ever-married. Details of survey methodology, sampling design, generation of sampling weights adjustments for non-response, and survey tools were published in the country report available on the Measure website. 

### 2.2. Study Variables

The standardized domestic violence module of LDHS, like other DHSs previously conducted in many countries around the world including African countries, entailed modified version of the Conflict Tactics Scale [7,15,20,21]. This scale was shown to have good validity and reliability in community as well as clinical settings [22,23]. 

### 2.3. Outcome Variable

Intimate partner violence (IPV) was defined as an ever-married respondent having ever experienced either emotional, physical, and/or sexual violence from a current or most recent husband/partner; with partner defined as cohabiting with a man as if married. IPV variable was derived from several LDHS questions and coded as a dichotomous outcome variable. Specifically, experience of physical violence was deemed extant if the respondent replied affirmatively to any of the following: push you, shake you, or throw something at you; slap you; twist your arm or pull your hair; punch you with his fist or with something that could hurt you; kick you, drag you, or beat you up; try to choke you or burn you on purpose; or threaten or attack you with a knife, gun, or any other weapon. Experience of emotional violence were derived from the affirmative answers to either: say or do something to humiliate you in front of others, threaten to hurt or harm you or someone close to you, or insult you or make you feel bad about yourself. While sexual violence experience was derived from affirmative answers to either: physically force you to have sexual intercourse with him even when you did not want to, physically force you to perform any other sexual acts you did not want to, or force you with threats or in any other way to perform sexual acts you did not want to. 

### 2.4. Explanatory Variables

Based on previous studies [6,7,8,9,10,11,12,13,14,15], 10 variables at the individual, husband/partner, and familial levels were studied for association with respondents having ever experienced intimate partner violence, i.e., women’s age, women’s educational attainment, women’s occupation, wealth index of the household/family, residential status in terms of urban and rural, number of living children, participation in decision making, acceptance of IPV, husband/partner’s use of alcohol, and having witnessed one’s father physically beating up one’s mother. Details on the derivation of each explanatory variable is provided as under:

Age: Respondents were asked about their age, based on their last birthday. LDHS datafile provides age in seven 5-year age groups, starting from 15–19 years and ending with the 45–49 years group. The age group 15–19 years was used as the reference category. 

Educational attainment: Respondents were grouped into four categories of either ‘no education’, ‘primary education’, ‘secondary education’, and ‘higher education’, i.e., more than secondary education. The ‘no education’ category was used as the reference category. 

Women’s occupation: Several occupational categories were specified in the LDHS. For this analysis, respondents were grouped into three categories of ‘professional, clerical, sales, services’; ‘does not work’; ‘agriculture self-employed, agriculture-employee, household and domestic work, skilled manual, and unskilled manual’. The ‘professional, clerical, sales, services’ category was used as the reference category. 

Wealth index of the family: LDHS gave each household scores based on the ownership of consumer goods that included television, bicycle/car, and housing attributes like toilet facilities, drinking water source, and type of flooring materials. Using principal component analysis, wealth quintiles were compiled and assigned to each household and its individual member. Five categories ranging from ‘poorest’, ‘poorer’, ‘middle’, ‘richer’, and ‘richest’ were calculated. The ‘poorest’ group was used as the reference category. 

Residential status: Respondent’s place of residence at the time of survey was grouped into two categories of ‘urban’ and ‘rural’ in the LDHS datafile. The ‘urban’ group was used as the reference category. 

Number of living children: Responses were coded as continuous variable in the LDHS, ranging from 0 to 15 children. For this analysis, four categories of ‘no/0 children’, ‘1–2 children’, ‘3–4 children’, and ‘5–15 children’ were created. The ‘zero children’ group was used as the reference category. 

Participation in decision making: Respondent having the latitude to make decisions, either alone or jointly with their husband/partner, in either of the three areas of healthcare seeking for herself, large household purchases, and visits to relatives was coded as having participated in the decision making. In LDHS, answers were coded into five groups of ‘respondent alone’, ‘respondent and partner/husband together’, ‘partner/husband alone’, ‘someone else’, and ‘other’. Not being able to participate either alone or jointly with husband/partner in any of the three areas was used as the reference category. 

Acceptance of intimate partner violence (IPV): Respondent replying affirmatively to believing that IPV is justified in either of the five scenarios of wife goes out without telling her partner/husband, neglects children, argues with partner/husband, refuses sex, and/or burns food, was coded as accepting of IPV. Not accepting all five scenarios was used as the reference category. 

Husband/partner’s alcohol use: Respondents were asked if their husband/partner drank alcohol and answers were either affirmative or negative. Negative answer was taken as the reference category. 

Witness parental IPV: Respondent replying affirmatively to the question on ever witnessing her father beat her mother. Negative answer was taken as the reference category. 

### 2.5. Statistical Analysis

Analyses was conducted in STATA version 17.1 (StataCorp, College Station, TX, USA) using the survey procedures to incorporate the complex sample design and sampling weight, accounting for the differential probability of selection. Missing data were not imputed; all hypothesis testing was 2-tailed, with statistical significance set at 2-sided *p* < 0.05. 

LDHS data set was downloaded as STATA format data file. As a first step, for outcome and all explanatory variables, unweighted counts, number of records with missing information, and cumulative weighted percentages were calculated. Secondly, simple binary logistic regression models were run to determine the statistical significance of every explanatory variable’s association with the outcome variable of having ever experienced IPV. Odds ratios, statistical significance, and 95% confidence intervals were calculated. Finally, all those explanatory variables that were found to be statistically significantly associated with the IPV were used in the final binary multiple logistic regression model. Adjusted odds ratios, statistical significance, and 95% confidence intervals were calculated for the final model. 

The association of all 10 explanatory variables with each sub-type of the outcome variable of IPV was examined next, using simple logistic regression models. Association of emotional, physical, and sexual violence was individually examined with each explanatory variable, in order to study the results stratified by each type of IPV. 

## 3. Results

Cumulatively, 1267 women reported one or more of the three types of violence. Emotional, physical, and sexual violence was reported by 968, 1046, and 175 women, respectively. While 136 women reported all three types of IPV, 756 women reported physical and emotional violence, 153 women reported physical and sexual violence, and 149 women reported both emotional as well as sexual violence. 

Table 1 shows the results of exploratory data analysis in terms of outcome and explanatory variables’ unweighted counts and cumulative weighted percentages, based on the 2331 ever-married women aged 15–49 years who were either currently or formerly in a union or living with a man, and completed the IPV questions on the domestic violence module of LDHS; the results pertain to their current or most recent husband/partner, with ‘partner’ defined as cohabiting with a man as if married. For 343 women, information on decision making in the areas of healthcare seeking for self, large household purchases, and visits to relatives was not available, as these questions were asked from only those women were currently, as opposed to formerly, in a union with a man or living with a man. 

Prevalence of having ever experienced emotional, physical, and/or sexual intimate partner violence perpetrated by either current husband or partner (if currently married) or most recent husband/partner (if divorced, separated, or widowed) was 55.29% (95% CI: 51.66–58.87) in women aged 15–49 years. While emotional, physical, or sexual IPV were reported by 41.76% (95% CI: 38.27–45.34), 44.80% (95% CI: 41.30–48.35), and 8.09% (95% CI: 6.32–10.30) women, respectively. The prevalence of having ever experienced physical and emotional IPV was 31.68 (95% CI: 28.83–34.67); physical and sexual IPV, 7.31% (95% CI: 5.53–9.62); emotional and sexual violence IPV, 6.82% (95% CI: 5.28–8.77); and 6.46% (95% CI: 4.93–8.42) reported all three types of IPV. The most common type of physical violence reported was ever having been slapped by husband/partner (41.49%); most common type of emotional violence reported was ever having been insulted or made to feel bad by husband/partner (34.18%); while the most common type of sexual violence reported was ever having been physically forced into unwanted sex by husband/partner (7.37%). The prevalence of having experienced physical and/or sexual violence was 45.57% (95% CI: 42.08–49.11).

Cumulatively, over half (55.06%) women were under the age of 35 years; 42.25% had no formal education; 24.76% did not work; 41.29% fell in the wealth index comprising of poorest and poorer; 54.90% were urban dwellers; 7.49% had no children; 89.08% made major decisions either alone or jointly with their husband/partner; 59.92% did not believe violence was acceptable; husband/partner’s use of alcohol was reported by 40.12%; and 75.38% did not witness or didn’t know if their father had ever physically beaten their mother. 

Table 2 shows the results of simple and multivariable logistic regression models in terms of crude odds ratios (OR), adjusted odds ratios (aOR), their statistical significance, and the associated 95% confidence intervals (CI). Out of the 10 explanatory variables examined in the bivariate analysis, 5 were found to be statistically significantly associated with having ever experienced any type of intimate partner violence. All these five explanatory variables, i.e., age, number of living children, acceptance of IPV, husband/partner’s use of alcohol, and having witnessed parental physical IPV, were added in the multivariable logistic regression model. As the results of this table show, with the exception of age, other four explanatory variables were found to be statistically significantly associated with the IPV in the multiple logistic regression model. 

In the final multivariable logistic regression model, women with 1–2 children experienced a reduction of 43.4% (aOR: 0.566; 95% CI: 0.337–0.951) in the odds of having experienced IPV compared with women with no living children. Odds that women experienced IPV were 1.89 times (95% CI: 1.412–2.522) higher in those who believed that IPV was justified, compared to women who believed that it was not so. Odds of IPV experience were 1.52 times (1.116–2.077) higher for women who had witnessed their father beat up their mother, compared to those who had not witnessed such abuse or did not know whether it took place. The odds of IPV were 2.89 times (2.243–3.788) higher in women whose husband or partner used alcohol, compared to those women whose husband or partner did not use alcohol. 

Since the outcome variable IPV was derived from three sub-factors of emotional, physical, and sexual violence. All the 10 explanatory variables were also analyzed with each of the 3 sub-factors of IPV, to study their individual relationships. The results of stratified analysis by type of violence are presented in Table 3 in terms of odds ratios and their statistical significance. The respondent’s place of residence and participation in decision making were not found to be statistically significantly associated with any of the three types of IPV. Use of alcohol by partner/husband was statistically significantly associated with all three types of IPV, individually. Acceptance of IPV and having witnessed parental IPV were individually associated with emotional as well as physical IPV. 

## 4. Discussion

Over half of the ever-married women aged 15–49 years reported having ever experienced one or more types of intimate partner violence perpetrated by their either current or most recent husband/partner, with the most common type being physical violence and the least common being sexual violence. The prevalence of having ever experienced both physical as well as emotional IPV was 31.68%, while the number of women who reported having ever experienced all three types of IPV was 6.48%. Having been slapped, insulted, made to feel bad, and physically forced into unwanted sex were the most common types of physical, emotional, and sexual IPV. The prevalence of having experienced physical and/or sexual violence was 45.57%, which is much higher than the prevalence for the WHO Africa region of 33% [5]. Based on the Liberia DHS 2019-20 country report available on the Measure website (www.measuredhs.com, accessed on 20 August 2021), two previous DHSs in Liberia were conducted in the years 2013 and 2007. The 2013 LDHS did not inquire about IPV, while the 2007 LDHS reported overall IPV prevalence of 49%; contrasting with 55% reported in LDHS 2019-20. Most respondents were under the age of 35 years, living in urban areas, not believing in acceptability of violence, and almost 90% made major decisions either alone or jointly with their partner/husband; over 40% had no formal education, and about a quarter had no job.

The bivariate analysis showed statistically significant association with IPV for respondent’s age, number of living children, acceptance of IPV, husband/partner’s use of alcohol, and having witnessed parental physical IPV. However, in multivariable analysis, age was not found to be statistically significantly associated with IPV. Although, the reported association between age and IPV is conflicting, with evidence of both younger as well as older age having higher association [6,11]. IPV association with women having children is reported to be high in women with higher number of children [8,11]. However, in this study, having 1–2 children bestowed protection to women from IPV in a statistically significant manner, compared with women with no living children. No statistical significance was found for women with 3 or more children and IPV. Acceptance of IPV by women was consistently shown to be associated with higher IPV reporting [9,13], and this was also borne out of this study. Alcohol use by one’s husband/partner was consistently associated with increased IPV experience by women [6,8,13], and this study reinforces this association. Finally, interparental violence determined by having witnessed one’s father physically beat up one’s mother was also associated with increased IPV [8,11,14], and the same association was found in this study as well. However, no statistically significant associations were found between IPV and urban/rural residency status or women’s educational attainment, despite two recent meta-analyses from sub-Saharan African countries reporting otherwise [7,15]. Similarly, employment status, wealth index, and participation in decision making were also not found to be statistically association with IPV in this study, contrary to other studies [6,9,10,11,13]. Communities marred by exposure to farrago of prolonged political conflicts and violence tend to increase IPV [17,18]. The absence of some associations found in this study perhaps reflects that pernicious influence. 

Based on the Liberia DHS 2019-20 country report, physical injuries resulting from IPV were sustained by 34% of ever-married women who reported having experienced physical or sexual IPV perpetrated by their current or most recent husband/partner. The inherent nature of cross-sectional survey design of LDHS preclude determination of any causal relationships, as only associations can be inferred. Secondly, by design, the LDHS only interviewed women 15–49 years of age, hence, older women are missed who might have had a higher proportion of having experienced IPV. Finally, the worst affected victims of IPV, the ones who lost their lives as a result of experiencing such violence, could not be factored into this analysis, i.e., healthy worker effect. Other limitations of the study include the fact that results are limited to ever-married women, and IPV perpetrated by the current or most recent husband/partner. Hence, lifetime IPV prevalence was not examined.

Higher rates of physical and mental health morbidities have been reported in the victims of IPV, including a wealth of literature about early childhood sexual abuse and mental and physical health outcomes in adulthood. However, owing to the cross-sectional nature of the survey, coupled with the fact that LDHS did not inquire about psychiatric morbidities, precludes the possibility of studying such sequalae in victims of IPV. 

Although association between IPV and women’s low educational attainment, not having a job or low occupational status, low family’s wealth index, and low participation in decision making were reported, results from LDHS did not bear them out. The results show that almost 90% of women did participate in major decisions, but it did not bestow protection from association with IPV. Furthermore, the IPV association in LDHS cuts across all groups of educational, occupational, wealth, and residency statuses. Failure of these explanatory variables in having discriminatory power in terms of association with IPV suggests more deeply entrenched IPV in the country. Thus, the need for better appreciation of human rights and equality of women in Liberia, coupled with health education efforts to address the menace of IPV, are required. 

The sub-factor analysis stratified by the three types of IPV revealed that respondent’s place of residence and participation in decision making were not found to be statistically significantly associated with any of the three types of IPV. Use of alcohol by partner/husband was statistically significantly associated with all three types of IPV. Acceptance of IPV and having witnessed parental IPV were individually associated with emotional as well as physical IPV. Identification of these three attributes and their strong associations with IPV reported in other studies using Demographic and Health Surveys data underscore the need for social and behavioral change communication, and policies for alcohol control in Liberia [24]. The need for more women empowerment and gender equality in Liberia is further underlined by the ‘Women Peace and Security Index’ that tracks 167 countries in the world for “sustainable peace through inclusion, justice, and security for women”; Liberia was ranked 144 in the 2019–2020 report [25].

## 5. Conclusions

This is the most recent nationally representative study on intimate partner violence of Liberian women where its correlates were also identified correlates were also identified in a multi-variable model. The lifetime physical and sexual IPV prevalence in ever partnered women aged 15–49 years was reported to be 27% globally, and 33% in WHO Africa region. In Liberia, 55.29% of ever-married women reported having experienced some form of IPV, including emotional violence perpetrated by the current or the most recent husband/partner. The most common type of IPV in Liberia was physical violence. The identified correlates of IPV highlights the need for promotion of self-esteem, social support for women, as well as strategies for empowerment and gender equality in Liberia.

## Figures and Tables

**Table 1:** Counts and proportions of study variables—Liberia DHS 2019–2020.

Variable	Unweighted Count	Cumulative
	(N = 2331)	Percentage
		(Weighted)
Outcome Variable		
Intimate Partner Violence	No = 1064	
(emotional, physical, and/or sexual)	Yes = 1267	55.29%
Emotional violence	No = 1363	
	Yes = 968	41.76%
Physical violence	No = 1285	
	Yes = 1046	44,80%
Sexual violence	No = 2156	
	Yes = 175	8.09%
Explanatory Variables		
Age	15–19y = 117	4.49%
	20–24y = 349	15.19%
	25–29y = 409	17.18%
	30–34y = 407	18.20%
	35–39y = 436	18.77%
	40–44y = 320	13.13%
	45–49y = 293	13.03%
Education	No education = 1139	42.25%
	Primary = 607	22.07%
	Secondary = 532	31.53%
	Higher = 53	4.15%
Occupation	Professional, clerical,	
	sales, services = 812	40.48%
	Does not work = 517	24.76%
	Agriculture	
	self-employed,	
	agriculture-employee,	
	household & domestic work,	
	skilled manual, and	
	unskilled manual = 998	34.77%
	Missing = 4	
Wealth	Poorest = 739	21.29%
	Poorer = 627	20.00%
	Middle = 497	21.38%
	Richer = 285	19.18%
	Richest = 183	18.15%
Residence	Urban = 795	54.90%
	Rural = 1536	45.10%
Children	0 = 127	7.49%
	1–2 = 730	34.72%
	3–4 = 757	31.69%
	5–15 = 717	26.10%
Decision making	Participated = 1791	89.08%
	Not participated = 197	10.92%
	* Not applicable = 343	
Acceptance	Not justified = 1310	59.92%
	Justified = 1021	40.08%
Alcohol use	No = 1406	59.88%
	Yes = 925	40.12%
Witnessed IPV	No = 1651	75.38%
	Yes = 680	24.62%

* Women who were formerly in a union or formerly living with a man were not asked this question.

**Table 2:** Crude odds ratios and adjusted odds ratios for all statistically significant associations between intimate partner violence and the selected variables—Liberia DHS 2019–2020.

Explanatory Variable	Unadjusted	*p*-Value	95% CI	Adjusted	*p*-Value	95% CI
		OR			OR	
Age						
15–19	Reference			Reference		
20–24	0.897	0.734	0.477–1.685	1.131	0.719	0.578–2.212
25–29	0.778	0.450	0.404–1.496	1.037	0.921	0.502–2.142
30–34	0.629	0.140	0.340–1.164	0.764	0.455	0.377–1.551
35–39	0.546	0.057	0.293–1.018	0.764	0.474	0.365–1.599
40–44	0.566	0.089	0.293–1.091	0.782	0.528	0.364–1.682
45–49	0.406	0.003	0.225–0.735	0.516	0.063	0.257–1.036
Education						
No Education	Reference			Not Applicable		
Primary	1.267	0.079	0.973–1.648			
Secondary	1.346	0.087	0.957–1.894			
Higher	0.945	0.896	0.404–2.209			
Occupation						
Professional,	Reference			Not Applicable		
clerical, sales,						
services						
Does not work	0.904	0.615	0.610–1.341			
Agriculture						
self-employed,						
agriculture-employee,						
household & domestic						
work, skilled manual,						
and unskilled manual	0.802	0.197	0.573–1.123			
Wealth						
Poorest	Reference			Not Applicable		
Poorer	0.901	0.484	0.674–1.206			
Middle	1.043	0.809	0.742–1.465			
Richer	1.332	0.145	0.906–1.959			
Richest	0.847	0.497	0.523–1.370			
Residence						
Urban	Reference			Not Applicable		
Rural	0.836	0.212	0.631–1.108			
Children						
No children	Reference			Reference		
1–2 children	0.527	0.010	0.323–0.859	0.566	0.032	0.337–0.951
3–4 children	0.503	0.008	0.302–0.838	0.632	0.126	0.351–1.137
5–12 children	0.413	0.001	0.246–0.694	0.568	0.069	0.309–1.046
Decision making						
Did not participate	Reference			Not Applicable		
Participated	0.876	0.599	0.534–1.437			
Acceptance						
Not justified	Reference			Reference		
Justified	2.068	< 0.0001	1.549–2.759	1.887	<0.0001	1.412–2.522
Alcohol use						
Does not use alcohol	Reference			Reference		
Uses alcohol	2.886	<0.0001	2.217–3.758	2.915	<0.0001	2.243–3.788
Witnessed IPV						
No	Reference			Reference		
Yes	1.830	<0.0001	1.381–2.425	1.523	0.008	1.116–2.077

OR = odds ratio.

**Table 3:** Odds ratios, significance levels, and 95% confidence intervals for the associations between three types of intimate partner violence with the selected variables—Liberia DHS 2019–2020.

Explanatory Variable	Emotional Violence		Physical Violence		Sexual Violence	
	OR (95% CI)	*p*-Value	OR (95% CI)	*p*-Value	OR (95% CI)	*p*-Value
Age						
15–19	Reference		Reference		Reference	
20–24	1.298 (0.712–2.367)	0.394	0.886 (0.475–1.653)	0.702	0.570 (0.185–1.760)	0.328
25–29	0.868 (0.490–1.538)	0.626	0.651 (0.347–1.220)	0.179	0.184 (0.068–0.495)	0.001
30–34	0.790 (0.457–1.366)	0.398	0.530 (0.287–0.982)	0.044	0.275 (0.095–0.0800)	0.018
35–39	0.689 (0.387–1.226)	0.205	0.504 (0.273–0.931)	0.029	0.344 (0.123–0.960)	0.042
40–44	0.950 (0.509–1.773)	0.872	0.498 (0.256–0.968)	0.040	0.363 (0.129–1.019)	0.054
45–49	0.674 (0.403–1.126)	0.131	0.300 (0.152–0.592)	0.001	0.244 (0.075–0.799)	0.020
Education						
No Education	Reference		Reference		Reference	
Primary	1.198 (0.848–1.692)	0.305	1.419 (1.106–1.820)	0.006	1.465 (0.958–2.240)	0.078
Secondary	1.141 (0.805–1.618)	0.459	1.400 (1.004–1.952)	0.047	0.803 (0.431–1.495)	0.488
Higher	0.768 (0.353–1.667)	0.503	0.914 (0.413–2.023)	0.823	0.321 (0.061–1.692)	0.179
Occupation						
Professional	Reference		Reference		Reference	
clerical, sales,						
services						
Does not work	0.762 (0.521–1.113)	0.159	1.213 (0.841–1.749)	0.301	2.481 (1.493–4.121)	<0.0001
Agriculture	0.936 (0.663–1.32)	0.707	0.822 (0.605–1.117)	0.209	1.768 (1.066–2.933)	0.027
self-employed,						
agriculture-employee,						
household & domestic						
work, skilled manual,						
and unskilled manual						
Wealth						
Poorest	Reference		Reference		Reference	
Poorer	0.926 (0.703–1.218)	0.581	0.916 (0.664–1.264)	0.593	1.442 (0.826–2.515)	0.197
Middle	0.931 (0.666–1.301)	0.675	1.159 (0.793–1.696)	0.445	1.213 (0.587–2.510)	0.601
Richer	0.828 (0.566–1.211)	0.328	1.235 (0.8000–1.908)	0.340	0.312 (0.146–0.666)	0.003
Richest	0.631 (0.397–1.003)	0.051	0.825 (0.495–1.373)	0.457	0.426 (0.177–1.030)	0.058
Residence						
Urban	Reference		Reference		Reference	
Rural	1.060 (0.802–1.399)	0.682	0.869 (0.660–1.445)	0.317	1.272 (0.746–2.169)	0.376
Children						
No children	Reference		Reference		Reference	
1–2 children	0.679 (0.385–1.98)	0.180	0.743 (0.406–1.359)	0.334	0.484 (0.187–1.249)	0.133
3–4 children	0.648 (0.365–1.151)	0.138	0.600 (0.324–1.112)	0.104	0.508 (0.198–1.306)	0.159
5–12 children	0.606 (0.338–1.085)	0.092	0.470 (0.261–0.847)	0.012	0.589 (0.261–1.326)	0.200
Decision making						
Did not participate	Reference	Reference			Reference	
Participated	0.760 (0.508–1.138)	0.182	0.775 (0.467–1.284)	0.321	0.357 (0.121–1.055)	0.062
Acceptance						
Not justified	Reference		Reference		Reference	
Justified	1.951 (1.448–2.628)	<0.0001	2.025 (1.553–2.642)	<0.0001	1.374 (0.815–2.315)	0.232
Alcohol use						
Does not use alcohol	Reference		Reference		Reference	
Uses alcohol	2.401 (1.863–3.094)	<0.0001	2.538 (1.924–3.347)	<0.0001	2.878 (2.071–3.999)	<0.0001
Witnessed IPV						
No	Reference		Reference		Reference	
Yes	1.967 (1.507–2.569)	<0.0001	1.561 (1.174–2.076)	0.002	1.266 (0.810–1.979)	0.299

OR = Odds Ratio.

## Data Availability

The approval for secondary analysis of LDHS was granted by Measure DHS using the online request form; datafile was downloaded from the Measure website www.measuredhs.com (accessed on 20 August 2021).
